# Influence of Parental Fatty Acid Desaturase 2 (*fads2*) Expression and Diet on Gilthead Seabream (*Sparus aurata*) Offspring *fads2* Expression during Ontogenesis

**DOI:** 10.3390/ani10112191

**Published:** 2020-11-23

**Authors:** Hanlin Xu, Shajahan Ferosekhan, Serhat Turkmen, Juan Manuel Afonso, María Jesús Zamorano, Marisol Izquierdo

**Affiliations:** 1 Aquaculture Research Group (GIA), Institute of Sustainable Aquaculture and Marine Ecosystems (ECOAQUA), Universidad de Las Palmas de Gran Canaria, Crta. Taliarte s/n, 35214 Telde, Spain; feroseaqua@gmail.com (S.F.); turkmen@uab.edu (S.T.); juanmanuel.afonso@ulpgc.es (J.M.A.); mariajesus.zamorano@ulpgc.es (M.J.Z.); marisol.izquierdo@ulpgc.es (M.I.); 2 ICAR-Central Institute of Freshwater Aquaculture, Bhubaneswar 751002, India; 3 Department of Biology, University of Alabama at Birmingham, Birmingham, AL 35294, USA

**Keywords:** aquaculture, lipid metabolism, embryogenesis, parental gene expression, parental nutritional status

## Abstract

**Simple Summary:**

The present study was on the gene expression of a rate-limiting enzyme in long chain polyunsaturated fatty acids (LC-PUFAs), fatty acyl desaturase 2 (*fads2*), throughout the embryonic development of a gilthead sea bream. The results showed a maternal transfer of *fads2* mRNA to the developing oocyte. The embryonic *fads2* expression might start after the neurula stage. No effect was found in *fads2* expression in developing eggs from broodstock fed with a diet rich in rapeseed oil or fish oil. The present study provides information on the change of LC-PUFA biosynthesis during embryogenesis.

**Abstract:**

Previous studies have shown that it is possible to increase the ability of marine fish to produce long-chain polyunsaturated fatty acid from their 18C precursors by nutritional programming or using broodstock with a higher fatty acyl desaturase 2 (*fads2*) expression. However, those studies failed to show the effect of these interventions on the expression of the *fads2* gene in the developing egg. Moreover, there were no studies on the temporal expression of the *fads2* during ontogeny in the gilthead sea bream (*Sparus aurata*). In order to determine the changes in expression of *fads2* during ontogeny, gilthead sea bream broodstock with a high (HRO) or low (LRO) *fads2* expression fed a diet previously used for nutritional programming, or a fish oil-based diet (LFO) were allowed to spawn. The samples were taken at the stages of spawning, morula, high blastula, gastrula, neurula, heart beating, hatch and 3 day-old first exogenous feeding larvae to determine *fads2* expression throughout embryonic development. The results showed the presence of *fads2* mRNA in the just spawned egg, denoting the maternal mRNA transfer to the developing oocyte. Later, *fads2* expression increased after the neurula, from heart beating until 3-day-old larvae, denoting the transition from maternal to embryonic gene expression. In addition, the eggs obtained from broodstock with high *fads2* expression showed a high docosahexaenoic acid content, which correlated with the downregulation of the *fads2* expression found in the developing embryo and larvae. Finally, feeding with the nutritional programming diet with the partial replacement of fish oil by rapeseed oil did not affect the long chain polyunsaturated fatty acid (LC-PUFA) contents nor *fads2* expression in the gilthead sea bream developing eggs.

## 1. Introduction

Long chain polyunsaturated fatty acids (LC-PUFAs), particularly docosahexaenoic acid (22:6n-3, DHA), eicosapentaenoic acid (20:5n-3, EPA) and arachidonic acid (20:4n-6, ARA), play relevant structural and functional roles in animal cells [1,2]. They are critical components of cell and organelle membranes [3] and their derived molecules, such as eicosanoids or docosanoids, participate in cell signaling processes [4]. In fish, LC-PUFAs are required for growth [5] or brain and immune system development and maintenance [6,7,8], among many other functions. In human nutrition, fish is the most important source of LC-PUFAs, especially DHA and EPA, whereas in farmed fish, fish oil (FO) is the traditional source of LC-PUFAs. However, FO is mostly derived from capture fisheries, which is a limited resource that would restrain the sustainable development of aquaculture. Thus, certain vegetable oils may partly replace FO in marine fish diets, since they are more environmentally and economically sustainable [9]. Despite vegetable oils lacking LC-PUFAs, they are abundant in their 18-carbon precursors [10].

The biosynthesis of LC-PUFAs is catalyzed by desturases and elongases. Like mammals, fish are unable to synthesize de novo LC-PUFAs, since they lack n-12 and n-15 desaturases, which only exist in some plants or invertebrates [11]. Therefore, either LC-PUFAs or their 18-carbon precursors must be included in diets for farmed fish. ARA and EPA are synthesized from 18:2n-6 and 18:3n-3, respectively, through the ∆6 desaturation–elongation and ∆5 desaturation pathway, or the elongation–∆8 desaturation and ∆5 desaturation pathway [12,13]. DHA may be synthesized in vivo from EPA through elongation–∆4 desaturation or elongation–elongation–∆6 desaturation and ß-oxidation [13]. Comparing with freshwater fish or euryhaline fish, marine fish have a lower ability to synthesize LC-PUFAs from their 18-carbon precursors [14]. One of the mechanisms leading to a limited ability from LC-PUFA synthesis in marine fish is the weak activity of these enzymes [15,16]. For instance, the activity of fatty acyl desaturase 2 (Fads2) in cod (*Gadus morhua* L.) is lower than in Atlantic salmon (*Salmo salar*) [17]. This could be related to the abundance of LC-PUFAs in the marine food web, originating from phytoplankton, which would reduce the need to synthesize these important fatty acids [18,19]. In addition, the LC-PUFA synthesis capacity of different fish species is also related to genome specificities. For instance, the fatty acyl desaturase 1 (*fads1*) gene, which codes for ∆5 desaturation, is absent in many telostei, although duplicated *fads2* or ∆5/∆6 bifunctional Fads2 may be found in those species [12,20]. Considering the absence of *fads1* in gilthead sea bream [12], *fads2* is a major rate-limiting enzyme in the LC-PUFA biosynthesis in this species. Indeed, in gilthead sea bream (*Sparus aurata*), Fads2 has both ∆6 and ∆8 desaturase activities [21]. On the contrary, ∆5 desaturate activity seems weak in gilthead sea bream, since the addition of ^14^C-labeled 18:3n-3 to the hepatocytes culture medium leads to a 41% of the radioactivity recovered in the ∆6 product 18:4n-3, whereas when ^14^C-labeled 18:4n-3 is added, only 0.7% of the radioactive is recovered from the Δ5 product, EPA [15].

Large variations in Fads2 activity are found among individuals of the same species in different environmental or nutritional conditions [6,22]. For instance, in gilthead sea bream, *fads2* expression in liver or red blood cells can be up to six or five times different among individuals from the same gender, when they are kept under the same environmental and nutritional conditions [23]. In addition, feeding a low LC-PUFA diet increases *fads2* expression or Fads2 products [18,22,24,25,26]. Therefore, either the individual selection of high *fads2* fish or feeding a low LC-PUFA diet could be used as a tool to produce fish with a higher Fads2 activity and thus, a better ability to synthesize LC-PUFAs when fed vegetable oil. On one hand, the offspring from broodstock with high *fads2* expression showed improved growth performance and feed utilization when challenged with a diet low in fishmeal (FM) and FO [27]. In addition, broodstock with high *fads2* expression showed a better reproductive performance, in terms of fecundity and sperm and egg quality [22]. On the other hand, feeding gilthead sea bream broodstock during the spawning season with a diet low in LC-PUFA diet improves the use of low FM/FO diets in their offspring [10,27] even up to 16 months [28]. In rainbow trout (*Oncorhynchus mykiss*), the nutritional condition by the complete replacement of dietary FM and FO by vegetable ingredients affects the expression of genes related to growth, amino acid and cholesterol metabolism [29], without negative effects for the second generation [30]. However, none of these studies were able to show any effect on the *fads2* expression in eggs due to the large variations obtained [22], which could be related to the different egg developmental stages in the eggs or the interference with a potential maternal transfer of *fads2* mRNA [31]. Despite that the *fads2* expression may occur at an early developmental stage in teleost [31,32,33], the temporal expression of *fads2* during the ontogenesis of gilthead sea bream has not yet been studied. Moreover, the effect of broodstock *fads2* expression and nutritional status on fads2 expression during ontogeny has also not been addressed.

Therefore, the aim of the present study was to determine, on the one hand, the temporal changes in *fads2* expression during ontogeny, and on the other hand, the influence of broodstock diet and the parental ability to express *fads2*. For that purpose, gilthead sea bream broodstock with a different ability to synthesize LC-PUFAs were fed either an FO-based diet or a diet previously used for the nutritional programming of progeny [22] and their eggs were sampled during ontogenesis to determine the temporal changes in *fads2* expression.

## 2. Materials and Methods

### 2.1. Ethical Statement

All the animal experiments were performed according to the European Union Directive (2010/63/EU) on the protection of animals for scientific purposes, at Fundación Canaria Parque Científico Tecnológico, University of Las Palmas de Gran Canaria (Las Palmas de Gran Canaria, Spain). REF: 007/2012 CEBA ULPGC.

### 2.2. Broodstock fads2 Expression

To obtain broodstock with a different ability to synthesize LC-PUFAs from the precursors linoleic acid (18:2n-6) and α-linolenic acid (18:3n-3), 3 months prior the spawning season, 185 gilthead sea bream brood fish of 1–2 kg were fed for one month at a diet high in linoleic acid and α-linolenic acid and low in LC-PUFAs promoting the upregulation of *fads2* [22]. Then, peripheral blood samples were collected and the *fads2* expression of blood cells was determined. Based on their *fads2* mRNA copy numbers per µL in blood cells, brood fish with the highest *fads2* expression values (H broodstock) or with the lowest ones (L broodstock) were chosen (Table 1). To determine the effect of the broodstock *fads2* expression on the *fads2* expression in developing eggs and larvae, 6 brood fish with high *fads2* expression (HRO) and 6 with low expression (LRO) were allocated into 4 × 1000 L tanks, (2 males and 1 female/tank) and fed a diet containing 1.76% FO and 7.54% rapeseed oil [22] (Supplement Table S1).

### 2.3. Effect of the Broodstock Diet

In order to determine the effect of the broodstock diet on the offspring *fads2* expression, another 3 low *fads2* expression broodstock (2 males and 1 female/tank) were fed with a diet containing FO as the only lipid source (LFO) and compared with the previous LRO broodstock. Both experimental diets were isoproteic and isolipidic and manufactured by Skretting ARC (Stavanger, Norway) [22] (Supplement Table S1). Compared with the FO diet, the replacement level of FO by vegetable oil in RO diet was 80%, a level that affects the offspring growth performance and lipid biosynthesis in response to a diet containing a low FM/FO diet [10,34]. In the present study, the content of LC-PUFAs (DHA + EPA + ARA) was 24.11% and 15.42% of total fatty acid in the FO diet and in RO diet, respectively (Supplement Table S2). All broodstock groups were manually fed twice per day (9:00 and 14:00 h) until apparent satiation. Seawater temperature was in the range of 18–22 °C (January–April 2018) and the fish were kept under a natural photoperiod (approximately 12 h light).

### 2.4. Sampling during Offspring Ontogenesis

**Figure 1:**
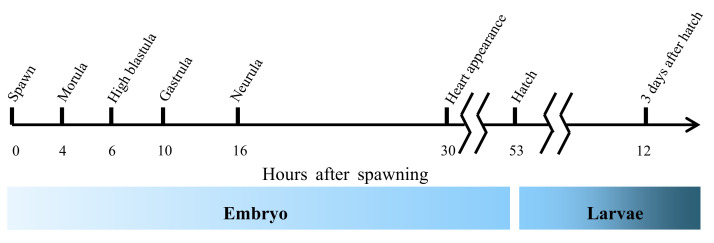
Timeline of the selected point during embryogenesis. The timeline is based on the description of stage during the embryogenesis of gilthead sea bream by Kamacı et al. 2005 [35] under experimental conditions.

At the end of the spawning season, eggs from the same spawning were collected and separately incubated in 500 L tanks. Eggs or larvae samples from each spawn were taken at consecutive stages of development: just after spawning (0 h post spawning (hps)), morula (4 hps), high blastula (6 hps), gastrula (10 hps), neurula (16 hps), heart beating (30 hps), hatch (53 hps) and 3-day-old larvae (125 hps) [35] (Figure 1). Samples were washed by diethyl pyrocarbonate water, kept overnight in RNAlater (MilliporeSigma, St. Louis, Burlington, MA, USA) at 4 °C and then stored at −80 °C until analysis.

### 2.5. RNA Extraction and Digital PCR

RNA from a 200 mg sample was extracted using TRI Reagent (MilliporeSigma, St. Louis, Burlington, MA, USA) and RNeasy kit (Qiagen, Hilden, Germany). RNAlater was removed from the samples before extraction. Then, each sample was weighed and 1 mL TRI Reagent with 1 steel bead was added. Then, the sample was homogenized in TissueLyser II (Qiagen, Hilden, Germany) at 30 Hz for 30 s and centrifuged at 13,000× *g* for 1 min. All of the liquid phase was transferred to a new 1.5 mL tube and 300 µL of chloroform (MilliporeSigma, St. Louis, Burlington, MA, USA) was added and mixed. The mixture was centrifuged at 13,000× *g* for 15 min. The transparent phase was isolated and mixed with the same volume of 70% ethanol. The mixture was loaded to a RNeasy mini spin column and washed by the RW1 and RPE offered in the kit according to the instruction of manufacturer. Concentrated RNA was eluted in 30 µL of RNase-free water. RNA quality was checked by 1.4% agarose electrophoresis and quantity was measured by Nanodrop 1000 (ThermoFisher, Waltham, MA, USA). Moreover, 1000 ng of RNA was used per sample for cDNA synthesis through iScript cDNA synthesis kit (Bio-Rad, Hercules, CA, USA) in iCycler (Bio-rad, Hercules, CA, USA). Digital PCR was conducted in QX200™ Droplet Digital™ PCR System (Bio-rad, Hercules, CA, USA). The reaction system, containing 100 ng of cDNA, *fads2* primer (forward: 5′-GCAGAGCCACAGCAGCAGGGA-3′, reverse: 5′-CGGCCTGCGCCTGAGCAGTT-3′, Gene bank No. GQ162822.1) and Evagreen SuperMix (Bio-rad) was loaded to the droplet generator to generate the oil droplet and then proceeded in C1000 Touch thermal cycler (Bio-Rad, Hercules, CA, USA). The cycling condition of PCR was: 95 °C for 5 min, followed by 40 cycles of 95 °C for 30 s, 63 °C for 1 min, and then stabilized the signal at 4 °C for 5 min, 90 °C for 5 min, finally the reaction was hold at 4 °C. The PCR amplification of the nucleic acid target in the droplets was read in a QX200 droplet reader (Bio-Rad, Hercules, CA, USA).

### 2.6. Lipid and Fatty Acid Analysis

Lipids from diets and 24 hps eggs were extracted with chloroform/methanol (2:1 *v*/*v*) [36] and transmethylated to obtain fatty acid methyl esters [37]. Then, fatty acid methyl esters were extracted with hexane:diethyl ether (1:1, *v*/*v*) and purified by the adsorption chromatography on NH2 Sep-pack cartridges (Waters, Milford, CT, USA). Afterwards, fatty acid methyl esters were separated by gas–liquid chromatography (GLC) (Agilent 7820A, Santa Clara, CA, USA) in a Supercolvax-10-fused silica capillary column (length: 30 mm, internal diameter: 0.32 mm, Supelco, Bellefonte, PA, USA) using helium as a carrier gas. The column temperature was 180 °C for the first 10 min, increasing to 215 °C at a rate of 2.5 °C min^−1^ and then held at 215 °C for 10 min [38]. Peaks were identified by comparison with external standards and well characterized FO (EPA 28, Nippai, Ltd. Tokyo, Japan).

### 2.7. Data Analysis

Target cDNA concentration was analyzed by QuantaSoft Analysis Pro tool (Bio-Rad, Hercules, CA, USA). The results were presented as the means ± standard deviation (S.D.). and analyzed by SPSS 20.0 (IBM, Armonk, NY, USA) for Mac. The comparison of the data between the different stages within the group were analyzed by one-way ANOVA. *Fads2* expression data between groups were analyzed by independent-samples t-test. Linear, exponential and logarithmic regression were performed through SPSS 20.0 (IBM, Armonk, NY, USA).

## 3. Results

### 3.1. Temporal Expression of fads2 during Offspring Ontogenesis

**Figure 2:**
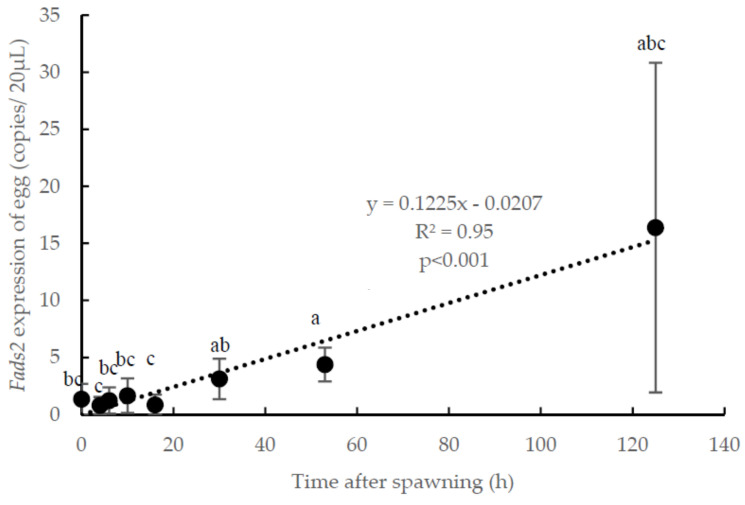
Temporal changes in *fatty acid desaturase 2* (*fads2*) mRNA copies during gilthead seabream (*Sparus aurata*) ontogenesis, from 0 to 125 h after spawning. Different letters denote significant differences among developmental stages (*p* < 0.05, *n* = 15). Bars bearing with different letter showed significant difference by one-way ANOVA analysis (*p* < 0.05). y, *fads2* expression of egg; x, time after spawning; R^2^, coefficient of determination.

Analysis of the average *fads2* expression values in eggs and larvae for all experimental groups showed an increase during embryogenesis, following a significant lineal regression (R^2^ = 0.95, *p* < 0.001) with time after spawning (Figure 2). Thus, *fads2* expression was registered at spawning (0 hps), remained low during the morula (4 hps), blastula (6 hps) and gastrula stages (10 hps) and even decreased until the neurula stage (16 hps), which showed the lowest *fads2* expression values (Figure 2). From the heart beating stage (30 hps) onwards, the *fads2* expression increased, being at this stage significantly (*p* < 0.05) higher than at the previous morula (4 hps) and neurula stages (16 hps), and at hatching (53 hps), it was also significantly (*p* < 0.05) higher than at the spawning to neurula stages (Figure 2). The highest values were observed in the 3-day-old larvae, although the large deviations did not allow obtaining significant differences with the *fads2* at other developmental stages (Figure 2).

### 3.2. Influence of Parental fads2 Expression in Offspring fads2 Expression during Ontogenesis

**Figure 3:**
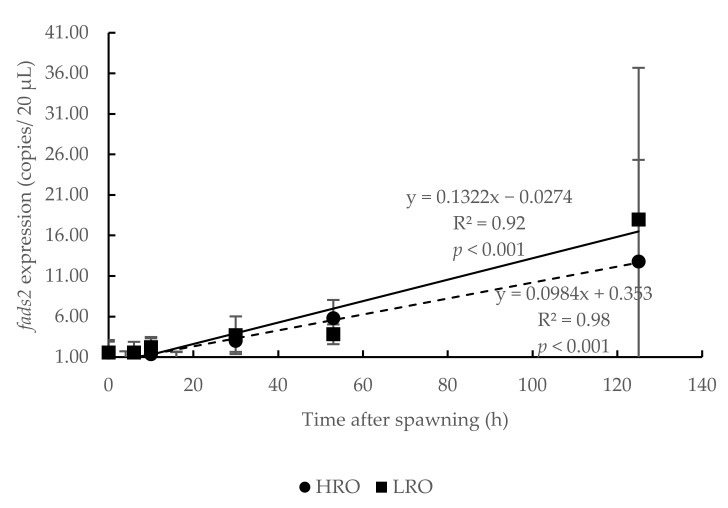
Influence of parental *fatty acid desaturase 2* (*fads2*) expression (high: HRO or low: LRO) on temporal changes in *fads2* mRNA copies during gilthead seabream (*Sparus aurata*) ontogenesis, from 0 to 125 h after spawning (*n* = 3). No significant difference was observed in the comparison using t-test (*p* > 0.05). y, *fads2* expression of egg; x, time after spawning; R^2^, coefficient of determination.

The *fads2* expression in eggs from broodstock with high (HRO) or low *fads2* expression (LRO) also followed significant (*p* < 0.001) lineal regressions (R^2^ = 0.98 and R^2^ = 0.92, respectively) (Figure 3). These regression lines crossed at 10 hps and thus, whereas in the earlier stages of development *fads2* expression tended to be higher for HRO, the later ones tend to be higher for LRO. Nevertheless, due to the large deviations among sampling batches, the t-test comparison between HRO and LRO values at each developmental stage did not showed significant (*p >* 0.05) differences (Figure 3).

**Figure 4:**
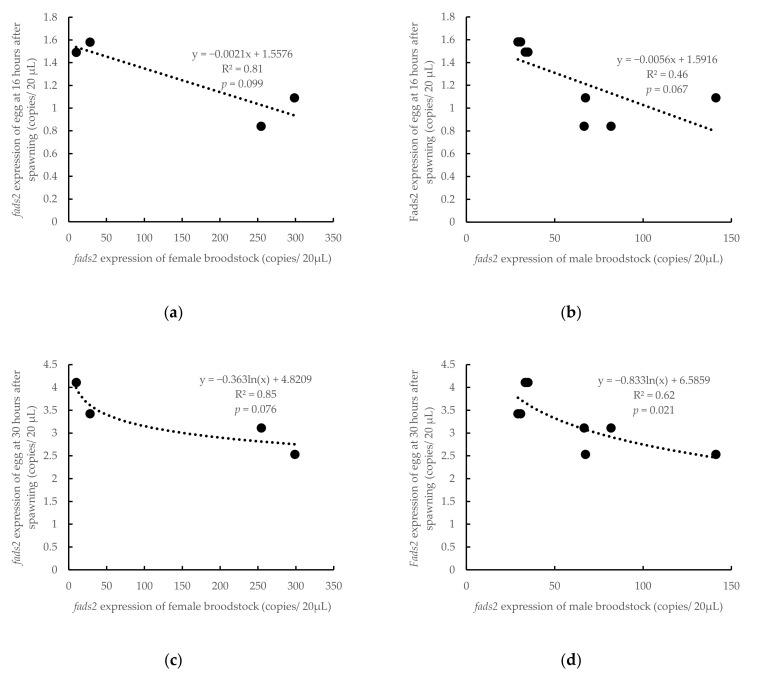
Relations of *fads2* mRNA copies between the egg at neurula stage (16 hps) and female (**a**) or male (**b**) broodstock (including both HRO and LRO broodstock), or between egg at the heart appearance stage (30 hps) and female (**c**) or male (**d**) broodstock. y, *fads2* expression of egg; x, *fads2* expression of broodstock; R^2^, coefficient of determination.

The *fads2* expression values in female and male broodstock were in the range of 0.49–14.92 and 1.26–7.06 copies/µL, respectively (Table 1). The comparison of broodstock and offspring *fads2* expression by regression analysis showed that the *fads2* expression at the neurula stage (16 hps) followed an inverse lineal relation with *fads2* expression in broodstock females (R^2^ = 0.81, *p* = 0.099) or males (R^2^ = 0.46, *p* = 0.067) (Figure 4). These regressions were higher and more significant at the heart beating stage (30 hps), when *fads2* expression in embryos followed an inverse logarithmic relation with *fads2* expression in females (R^2^ = 0.85, *p* = 0.076) or males (R^2^ = 0.62, *p* = 0.021) (Figure 4).

### 3.3. Influence of Parental fads2 Expression in Egg Fatty Acid Profiles

At 24 hps, the DHA content in eggs obtained from the HRO group was 6.46% higher than that from LRO group (*p* = 0.028). In addition, the ratio between DHA and ARA in the eggs of the HRO group was 8.97 higher than in eggs of the HFO group (*p* = 0.004). The content of two very long chain n-6 PUFAs, 22:4n-6 and 22:5n-6, were significantly lower in the HRO group than in the LRO group (*p* < 0.05). Similar differences were also found in the comparison in 18:0, 18:2n-4 and 20:2n-9 content between two groups (*p* < 0.05) (Table 2). The integrated fatty acid composition of egg was shown in Supplement Table S3.

**Figure 5:**
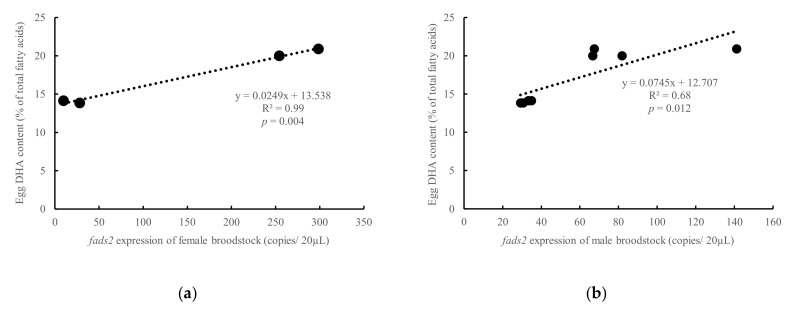
Relations between the DHA contents in the egg at 24 h after spawning and *fads2* mRNA copies of female (**a**) or male (**b**) broodstock (including both HRO and LRO broodstock). y, DHA content is eggs; x, *fads2* expression of broodstock; R^2^, coefficient of determination.

**Figure 6:**
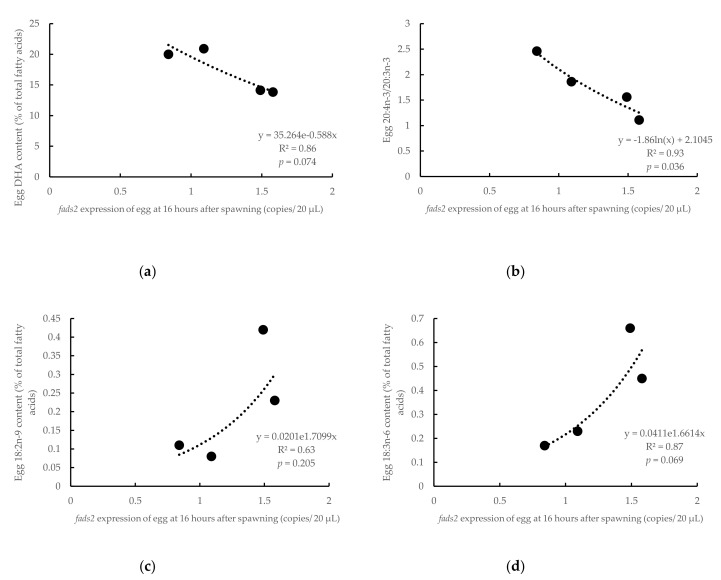
Relations between the *fads2* mRNA copies of egg at 16 h after spawning and the content of DHA (**a**), ratio 20:4n-3/20:3n-3 (**b**), 18:2n-9 (**c**) and 18:3n-6 (**d**) in eggs at 24 h after spawning (including eggs from both HRO and LRO broodstock). y, respective fatty acids in eggs at 24 h after spawning; x, *fads2* expression of egg at 16 h after spawning; R^2^, coefficient of determination.

**Figure 7:**
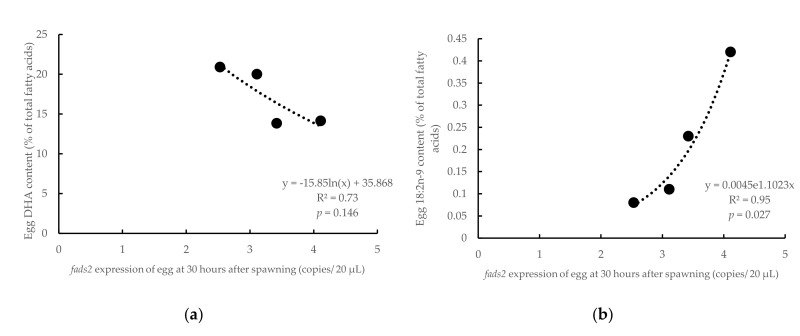

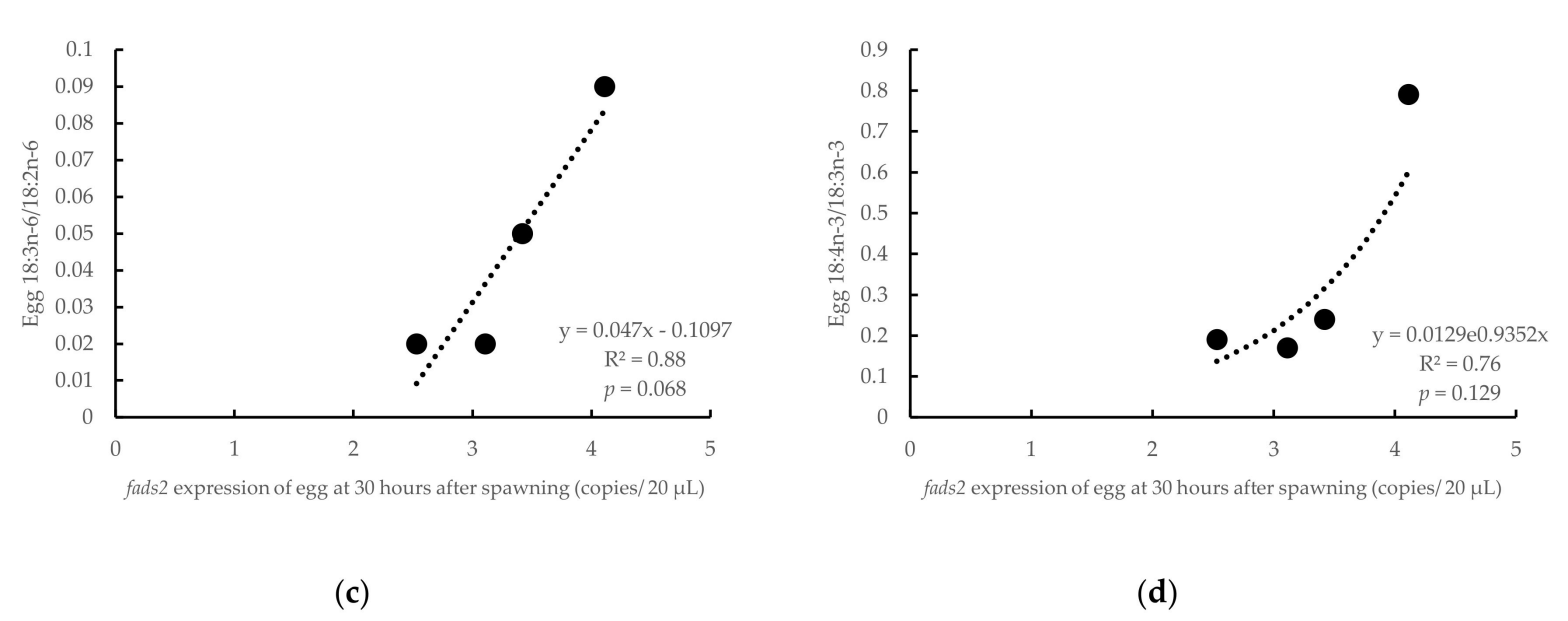
Relations between *fads2* mRNA copies of egg at 30 h after spawning with the content of DHA (**a**), 18:2n-9 (**b**), ratio 18:3n-6/18:2n-6 (**c**) and 18:4n-3/18:3n-3 (**d**) in eggs at 24 h after spawning (including eggs from both HRO and LRO broodstock). y, respective fatty acids in eggs at 24 h after spawning; x, *fads2* expression of eggs at 30 h after spawning; R^2^, coefficient of determination.

The *Fads2* expression in both female and male broodstock was significantly (*p* < 0.05) and positively related to the DHA contents in the eggs (R^2^ = 0.99, *p* = 0.004 and R^2^ = 0.68, *p* = 0.012, for females and males, respectively) (Figure 5). In comparison, the DHA content in eggs was inversely related (R^2^ = 0.86, *p* = 0.074) to the *fads2* expression in the eggs at neurula stage (16 hps) (R^2^ = 0.86, *p* = 0.07, Figure 6a). Similarly, the ratio 20:4n-3/20:3n-3, an indicator of desaturase activity, also followed a significant (R^2^ = 0.93, *p* = 0.036) negative relation with *fads2* expression (Figure 6b). On the contrary, 18:2n-9, the product of Fads2 from 18:1n-9, contents in eggs were positively related to the *fads2* expression in egg at the neurula stage (16 hps) (R^2^ = 0.63, *p* = 0.205) (Figure 6c). In addition, 18:3n-6, a product of Fads2 on 18:2n-6 was directly related to the *fads2* expression in the egg at neurula stage (16 hps) (*p* < 0.10) (Figure 6d). When the eggs reached the heart appearance stage (30 hps), the DHA contents in eggs were inversely related to the *fads2* expression in eggs (R^2^ = 0.73, *p* = 0.146), which was similar to the correlation at the neurula stage (Figure 7a). However, in contrast to the relation at the neurula stage, 18:2n-9 contents in eggs were directly related to the *fads2* expression in the egg at the heart beating stage (30 hps) (R^2^ = 0.95, *p* = 0.027) (Figure 7b). The ratios between precursors and products of ∆6 desaturation, 18:3n-6/18:2n-6 and 18:4n-3/18:3n-3, directly related to the *fads2* expression in the eggs (Figure 7c,d).

### 3.4. Influence of Broodstock Diet in Offspring fads2 Expression during Ontogenesis

**Figure 8:**
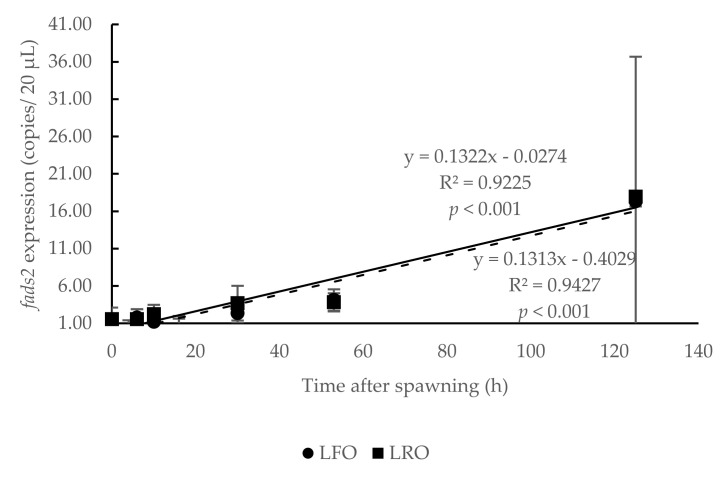
Changes in *fads2* expression during gilthead seabream (*Sparus aurata*) ontogenesis, from 0 to 125 h after spawning (*n* = 3) from broodstock with a low expression of *fads2* and fed either a FO (LFO) or a RO (LRO) diet during spawning. y, *fads2* expression of egg; x, time after spawning; R^2^, coefficient of determination.

No significant (*p >* 0.05) difference was observed on *fads2* expression during embryogenesis between eggs from broodstock fed with a diet rich in FO (LFO broodstock) or RO (LRO broodstock), which followed a similar positive linear regression (*p* < 0.001) (Figure 8).

## 4. Discussion

The present study aimed to determine the temporal changes of *fads2* expression during the ontogeny of gilthead seabream and the potential influence of broodstock diet and the parental ability to express *fads2*. Overall, the results showed the presence of *fads2* mRNA even in just spawned egg, denoting the maternal mRNA transfer to the developing oocyte. Then, from the neurula stage onwards, *fads2* expression increased, denoting the transition from maternal to embryonic gene expression. Regarding the broodstock, eggs obtained from parents with high *fads2* expression showed a high DHA content, together with the downregulation of *fads2* expression. Finally, the partial replacement of FO by RO did not affect LC-PUFA contents nor *fads2* expression in gilthead sea bream eggs.

### 4.1. Changes of fads2 mRNA in Gilthead Seabream Eggs during Ontogeny

The use of gilthead seabream broodstock with a high ability to express *fads2* when fed low FO diets improves reproductive success [22]. However, their consequences on the *fads2* expression in the offspring, particularly during the development of embryo and larvae, have not been yet studied. This is partly due to the lack of knowledge on the ontogeny of *fads2* expression. The present study has demonstrated that in gilthead seabream, *fads2* expression can be detected even in the just spawned egg (0 hps) immediately after fertilization. These results are in agreement with the presence of fatty acid desaturase gene (*fad*) expression in zebrafish zygote [31] and denote a maternal mRNA transfer to the developing oocyte. Despite that maternal mRNA transfer would be proven by the presence of transcripts in unfertilized eggs, no differences were found in mRNA expressions between unfertilized and 1.5 h post-fertilized zebrafish eggs, denoting that just fertilized or just spawn eggs are useful to study maternal transfer mRNAs [39]. In fish, maternal mRNAs produced by the mother based on her genome are incorporated into the developing oocytes at very early stages of oogenesis [40]. Despite maternal transcripts may originally be distributed throughout the oocyte cytoplasm, after fertilization most of them are pulled into the blastodisc through cytoplasmic streaming or ooplasmic segregation [41,42]. There, from mid-blastula on-wards, maternal mRNAs become important components of the protein translation machinery contributing to the proper embryogenesis, and their expression is being regulated by their stability and degradation [40]. In seabream, *fads2* mRNA copies were constant during morula (4 hps), blastula (6 hps) and gastrula stages (10 hps) and decreased at the neurula stage (16 hps) suggesting the degradation of the maternal *fads2* mRNA at this stage.

From the neurula stage onwards, a significant increase was observed in the seabream *fads2* expression, denoting the transition from maternal to embryonic gene expression. At the neurula stage the embryo has already established the bases for the development of the principal organs and systems, including neural and circulatory systems, and the increased *fads2* expression may contribute to fulfill the LC-PUFA requirements for organ development [6]. LC-PUFAs, and specially DHA, are necessary for brain development and functioning in seabream as in other vertebrates [43]. In agreement with this, *fad* is particularly expressed in the head area of zebrafish [31]. Indeed, *fads2* expression in gilthead seabream was significantly upregulated at heart beating, when brain development becomes very notorious, and remained increasing during the hatching and 3-day-old larvae stages. These results were relatively different from those obtained in zebrafish, where *fad* expression is claimed to increase earlier during embryo development, from 12 h post fertilization onwards [31]. Nevertheless, these conclusions in zebrafish are based on comparisons of transcript levels from RT-PCR analyses and have to be made cautiously, as stated by the authors [31], whereas the present study was based on digital PCR determining absolute quantification mRNA copies with nine replicates for each developmental stage. Indeed, rather than at 12 h, a more evident increase in *fad* expression was observed at 19 h post fertilization [31], coinciding with the late segmentation stage in zebrafish when organogenesis occurs [44], in agreement with our findings in gilthead seabream. Therefore, a marked upregulation of the embryo *fads2* expression was found after the neurula stages, particularly from heart beating, suggesting that the determination of *fads2* expression in the offspring embryo should be conducted from this stage onwards to prevent the potential influence of the maternal mRNA.

### 4.2. Influence of Parental fads2 Expression in Offspring fads2 Expression during Ontogenesis

The regression lines for the temporal expression of *fads2* during the ontogenesis of seabream offspring from high or low *fads2* expressing broodstock crossed at 10 hps, denoting a higher number of mRNA copies in offspring from high *fads2* expressing broodstock. This observation would be in agreement with the maternal transfer of *fads2* mRNA previously discussed. Interestingly, in later developmental stages, the *fads2* expression tended to be lower in offspring from broodstock with high *fads2* expression, as it was supported by the significant negative relation between *fads2* expression in broodstock and offspring at neurula or, particularly, heart beating stages. This downregulation of *fads2* expression in offspring from broodstock with higher *fads2* expression, seemed to be due to their increased DHA content in the eggs in comparison to those from lower *fads2* expression broodstock. The maternal transfer of DHA into fish eggs may occur during endogenous vitellogenesis by the synthesis in the ovaries of lipid vesicles that will lead to the oil globule, or exogenous vitellogenesis by the synthesis of vitellogenin in the liver forming the lipoprotein yolk lipids [45,46]. In gilthead seabream broodstock, *fads2* is expressed in both tissues, but specially in the liver, where it increases the production of LC-PUFAs, particularly, DHA [22]. Whereas in seabream male broodstock, DHA in liver increases proportionally to the hepatic expression of *fads2*, in the females DHA does not accumulate in the liver [22], but it is incorporated into vitellogenin and transferred into the developing oocytes. In agreement, in the present study, DHA contents in the eggs were positively correlated to the *fads2* expression in the broodstock, particularly females, denoting the maternal transfer of DHA. DHA is highly demanded during embryogenesis and larval development to sustain growth, being the most abundant fatty acid in the eggs of many fish species [19,47]. DHA can resist membrane packing, regulate membrane fluidity and has a structural function on the cell membrane, directly promoting growth [6]. In addition, it is a precursor of docosanoids, which function as local hormones (autacoids) with autocrine and paracrine functions targeting cells in the area where they are formed, and are involved in the regulation of a wide array of cellular pathways and cascades, including cell proliferation and differentiation [6].

In turn, increased DHA contents in the seabream eggs were inversely related to *fads2* expression in embryos at neurula and heart appearance stages, denoting the downregulation of *fads2* expression by DHA as observed during the whole fish life cycle [25,48,49]. Indeed, feeding a FO diet with high levels of LC-PUFAs, particularly DHA, inhibits *fads2* expression in comparison to a vegetable oil diet low in these fatty acids [26,48,49,50]. In mammals, the presence of LC-PUFAs restricts the level *FADS2* through the E-box like sterol regulatory element [51]. Studies on the compartmental location of fatty acids during fish ontogeny have shown that despite DHA is initially found in the yolk, this fatty acid is preferentially incorporated into structural lipids in the embryo tissues [52], where it would downregulate *fads2* expression. Thus, the higher the *fads2* expression in the broodstock, the higher the incorporation of DHA into the eggs, and as such the increased incorporation of DHA inhibited the *fads2* expression in the eggs.

In agreement, the eggs obtained from low *fads2* parents showed low DHA contents and a high *fads2* expression, associated with the increased ∆6-activity on n-3 and n-6 precursors as denoted by the increase in the ratio of synthesis from 18:3n-6 to 18:2n-6, or from 18:4n-3 to 18:3n-3. In addition, high *fads2* expression in the egg was also associated to a reduction in ∆8-desaturase activity as denoted by the reduction in the ratio of synthesis of 20:4n-3 from 20:3n-3. The ∆8-desaturase activity is an alternative but a minor pathway for LC-PUFA biosynthesis, more active when there is a high demand of eicosanoid synthesis [21,53]. The study of this alternate pathway in baker’s yeast (*Saccharomyces cerevisiae*) with a mammals *FADS2* gene showed that the activity of ∆6-desaturase is higher than that of ∆8-desaturase by 7.2-fold on n-6 fatty acids and by 23-fold on n-3 fatty acids [53]. In agreement, the negative correlation of the ∆8 activity and *fads2* expression in gilthead seabream embryos could be related with the activation of ∆6 activity of Fads2 in the embryos with lower DHA content.

### 4.3. Influence of Broodstock Diet in Offspring fads2 Expression during Ontogenesis

FO has been partly replaced by RO in broodstock diets, in order to induce nutritional programing in the offspring for a better utilization of low FM and low FO diets [27]. Since dietary LC-PUFAs, high in FO, are required for good spawning quality in fish [47], FO content in broodstock diets can be replaced by RO for nutritional programing purposes, but not as much as to reduce spawning quality [10]. However, their effect in the temporal expression of *fads2* during gilthead seabream ontogenesis has not been studied. In the present study, feeding the same broodstock diets as in previous studies [22] did not affect *fads2* expression during embryogenesis. Even though the dietary LC-PUFA levels were slightly different, the LC-PUFA contents in the eggs were similar. Despite the egg contents in LC-PUFAs being usually correlated to the LC-PUFA levels in broodstock diets, there is a preferential retention of these fatty acids in the eggs, particularly in species with oligolecythic eggs, such as gilthead seabream and therefore, small dietary differences may not be reflected in the fatty acid profiles of the eggs [47,54]. Moreover, the optimum LC-PUFA content of gilthead sea bream broodstock during spawning season is 11.27% of the total fatty acid in the diet [54] and both broodstock diets in the present study fulfill these requirements. Thus, the similarity of the LC-PUFA content in eggs can explain why there was no difference on the *fads2* expression in eggs from the broodstock fed different lipid sources. These results agree well with the lack of effect of these diets on *fads2* expression in gilthead seabream eggs [22]. However, more extreme diets may affect the expression of LC-PUFA biosynthesis-related genes. For instance, in *Solea senegalensis*, feeding broodstock with a diet markedly higher in LC-PUFAs suppresses *elovl5* expression in eggs [55]. Further studies with diets containing extreme levels of LC-PUFAs should be conducted to understand the effect of broodstock diets on the expression of genes related to LC-PUFA biosynthesis.

## 5. Conclusions

In summary, the results of the present study showed the presence of *fads2* mRNA in the just spawned gilthead seabream egg, denoting the maternal mRNA transfer to the developing oocyte, whereas from the neurula stage onwards, the *fads2* expression increased, denoting the transition from maternal to embryonic gene expression. In addition, the eggs obtained from broodstock with high *fads2* expression showed a high DHA content, which could be responsible for the downregulation of *fads2* expression in the developing embryo and larvae. Finally, the partial replacement of FO by rapeseed oil did not affect LC-PUFA contents nor *fads2* expression in gilthead seabream eggs.

## Figures and Tables

**Table 1:** Average *fads2* expression in the peripheral blood cells of the broodstock individuals used in the present study.

Group	Gender	*fads2* Expression (Copies/μL)
HRO (High *fas2* + RO diet)	Female 1	14.92
	Male 1-1	7.06
	Male 1-2	3.37
	Female 2	12.72
	Male 2-1	4.09
	Male 2-2	3.33
LRO (Low *fas2* + RO diet)	Female 3	1.41
	Male 3-1	1.53
	Male 3-2	1.46
	Female 4	0.49
	Male 4-1	1.74
	Male 4-2	1.66
LFO (low *fas2* + FO diet)	Female 5	1.11
	Male 5-1	1.26
	Male 5-2	1.27

FO, fish oil; RO, rapeseed meal.

**Table 2:** Main fatty acids of eggs at 24 hps from broodstock fed an RO diet during spawning and showing either a high (HRO) or low (LRO) expression of *fads2.*

Fatty Acids	HRO		LRO	
	Mean	S.D.	Mean	S.D.
18:1n-9	27.06	1.03	19.69	7.04
18:2n-9	0.10	0.02	0.33	0.13
18.2n-6	10.81	0.24	8.63	1.76
18:3n-6	0.20	0.04	0.56	0.15
18:3n-3	2.92	0.12	2.20	0.53
18:4n-3	0.53	0.01	1.02	0.58
20:4n-6	0.76	0.03	0.78	0.01
20:5n-3	5.95	0.10	6.98	3.49
22:4n-6	0.08	0.02	0.63 *	0.07
22:5n-6	0.25	0.04	0.60 *	0.04
22:6n-3	20.45 *	0.64	13.99	0.21
SFA	16.76	0.36	25.38 *	0.31
MUFA	35.74	0.82	31.76	5.30
PUFA	47.49	1.20	42.87	4.97
n-3 FA	33.84	0.83	28.01	5.15
n-6 FA	12.67	0.38	12.06	1.42
DHA/ARA	26.91 *	0.16	17.94	0.05
DHA/EPA	3.44	0.05	2.28	1.11

* denotes the significant difference between the two groups (*p* < 0.05). ARA, arachidonic acid; DHA, docosahexaenoic acid; EPA, eicosapentaenoic acid; FA, fatty acid; SFA, saturated fatty acid; MUFA, monounsaturated fatty acid; PUFA, polyunsaturated fatty acid; S.D. standard deviation.

## Supplementary Materials

The following are available online at https://www.mdpi.com/2076-2615/10/11/2191/s1, Table S1: Ingredients of the experimental diets used to feed broodstock during the spawning season, Table S2: Fatty acid composition of the experimental diets used to feed broodstock during the spawning season, Table S3: Fatty acid composition of eggs at 24hps from broodstock fed a RO diet during spawning and showing either high (HRO) or low (LRO) expression of *fads2*.

## Abbreviations

LC-PUFAs	long chain polyunsaturated fatty acids
DHA	docosahexaenoic acid
EPA	eicosapentaenoic acid
ARA	arachidonic acid
FO	fish oil
fads	fatty acyl desaturase
FM	fishmeal
hps	hour post spawning
SFA	saturated fatty acid
MUFA	monounsaturated fatty acid
S.D.	standard deviation
