# Supplementary material for: Influence of Parental Fatty Acid Desaturase 2 (fads2) Expression and Diet on Gilthead Seabream (Sparus aurata) Offspring fads2 Expression during Ontogenesis
Source: Animals (Basel). 2020 Nov 23;10(11):2191. doi: 10.3390/ani10112191 (PMC7700513; doi:10.3390/ani10112191)
Supplement: Supplementary file 1 [file animals-10-02191-s001.pdf]

animals

MDPI

Article

Influence of Parental *fatty acid desaturase 2* (*fads2*) Expression and Diet on Gilthead Seabream (*Sparus aurata*) Offspring *fads2* Expression during Ontogenesis

Hanlin Xu [1,*], Shajahan Ferosekhan [1,2], Serhat Turkmen [1,3], Juan Manuel Afonso [1], María Jesús Zamorano [1] and Marisol Izquierdo [1]

[1] Aquaculture Research Group (GIA), Institute of Sustainable Aquaculture and Marine Ecosystems (ECOAQUA), Universidad de Las Palmas de Gran Canaria, Crta. Taliarte s/n, 35214 Telde, Spain; feroseaqua@gmail.com (S.F.); turkmen@uab.edu (S.T.); juanmanuel.afonso@ulpgc.es (J.M.A.); mariajesus.zamorano@ulpgc.es (M.J.Z.); marisol.izquierdo@ulpgc.es (M.I.)

[2] ICAR-Central Institute of Freshwater Aquaculture, Bhubaneswar, 751002, India

[3] Department of Biology, University of Alabama at Birmingham, Birmingham, AL 35294, USA

* Correspondence: hanlin.xu101@alu.ulpgc.es

Received: 17 October 2020; Accepted: 19 November 2020; Published: 23 November 2020

Table 1. Ingredients of the experimental diets used to feed broodstock during the spawning season [1].

Ingredients (%)	FO	RO
Fish meal (North-Atlantic 12C)	59.36	59.36
Squid meal	3.00	3.00
Krill meal	7.00	7.00
Wheat	20.57	20.57
Fish oil (South American)	9.30	1.76
Rapeseed oil	0.00	7.54
Vitamin-mineral premix *	0.50	0.50
L-Histidine HCl	0.27	0.27
Proximate composition		
Crude protein (%DM)	53.4	54.6
Crude lipid (%DM)	18.8	17.3
Ash (%DM)	11.3	11.6
Moisture (%)	7.9	7.3

FO, fish oil rich diet; RO, rapeseed oil rich diet. Vitamin-mineral premix *: vitamins (mg/kg): A 3.8, D 0.05, E 102.4, K3 9.8, B1 2.7, B2 8.3, B6 4.8, B12 0.25, B3 24.8, B5 17.2, folic acid 2.8, H 0.14, C 80; minerals (mg/kg): cobalt 0.94, iodine 0.7, selenium 0.2, iron 32.6. manganese 12, copper 3.2, zinc 67; other (g/kg): taurine 2.45, methionine 0.5, histidine 1.36, cholesterol 1.13. DSM, (Netherlands), Evonik (Germany), Deutsche Lanolin Gesellschaft (Germany).

Table 2. Fatty acid composition of the experimental diets used to feed broodstock during the spawning season [1].

Fatty acid (% of total fatty acid)	FO	RO
14:0	5.04	1.87
14:1n-5	0.15	0.08
15:0	0.46	0.17
16:0 ISO	0.09	0.09
16:0	18.83	9.42
16:1n-7	6.84	2.67

16:1n-5	0.26	0.11
16:2n-4	0.75	0.29
17:0	0.83	0.20
16:3n-4	0.23	0.17
16:3n-1	0.20	0.11
16:3n-3	0.12	0.08
16:4n-3	1.09	0.43
18:0	3.95	2.47
18:1n-9	12.82	31.76
18:1n-7	3.37	3.28
18:1n-5	0.30	0.16
18:2n-9	0.19	0.04
18:2n-6	4.11	11.14
18:2n-4	0.24	0.09
18:3n-6	0.32	0.13
18:3n-4	0.15	0.14
18:3n-3	1.30	4.95
18:4n-3	2.19	1.22
18:4n-1	0.00	0.11
20:0	0.47	0.61
20:1n-9	3.77	4.06
20:1n-7	0.31	0.18
20:2n-9	0.06	0.05
20:2n-6	0.20	0.17
20:3n-9	0.07	0.09
20:3n-6	0.12	0.10
20:4n-6	1.04	0.43
20:3n-3	0.15	0.12
20:4n-3	0.57	0.35
20:5n-3	11.96	6.57
22:1n-11	3.73	4.98
22:1n-9	0.51	0.66
22:4n-6	0.17	0.23
22:5n-6	0.43	0.27
22:5n-3	1.40	0.79
22:6n-3	11.11	8.42

Table S3. Fatty acid composition of eggs at 24hps from broodstock fed a RO diet during spawning and showing either high (HRO) or low (LRO) expression of *fads2*.

**Title**	**HRO**		**LRO**	
	**Mean**	**S.D.**	**Mean**	**S.D.**
14:0	1.12	0.08	4.03	1.94
14:1n-7	0.02	0.01	0.13	0.02
14:1n-5	0.05	0.01	0.18	0.11
15:0	0.17	0.00	0.38	0.11
15:1n-5	0.03	0.01	0.18	0.04
16:0 ISO	0.04	0.00	0.18	0.02
16:0	11.58	0.25	16.17	2.02
16:1n-7	2.70	0.07	3.73	0.52
16:1n-5	0.07	0.02	0.27	0.04
16:2n-4	0.19	0.00	0.33	0.17
17:0	0.14	0.02	0.26	0.09
16:3n-4	0.18	0.01	0.49	0.35
16:3n-3	0.11	0.01	0.40	0.29
16:3n-1	0.10	0.01	0.34	0.30
16:4n-3	0.14	0.03	0.37	0.24
18:0	3.58	0.06	4.05 *	0.08

18:1n-9	27.06	1.03	19.69	7.04
18:1n-7	3.09	0.04	4.98	1.90
18.1n-5	0.15	0.01	0.82	0.74
18:2n-9	0.10	0.02	0.33	0.13
18.2n-6	10.81	0.24	8.63	1.76
18:2n-4	0.11	0.01	0.31 *	0.03
18:3n-6	0.20	0.04	0.56	0.15
18:3n-4	0.11	0.00	0.36	0.08
18:3n-3	2.92	0.12	2.20	0.53
18:4n-3	0.53	0.01	1.02	0.58
18:4n-1	0.09	0.01	0.20	0.07
20:0	0.15	0.01	0.33	0.08
20:1n-9	0.24	0.01	0.23	0.01
20:1n-7	1.50	0.14	0.77	0.44
20.1n-5	0.19	0.01	0.22	0.06
20:2n-9	0.08	0.01	0.22 *	0.02
20:2n-6	0.42	0.06	0.41	0.02
20:3n-9	0.05	0.01	0.24	0.13
20:3n-6	0.17	0.01	0.47	0.26
20:4n-6	0.76	0.03	0.78	0.01
20:3n-3	0.31	0.06	0.37	0.01
20:4n-3	0.65	0.01	0.49	0.10
20:5n-3	5.95	0.10	6.98	3.49
22:1n-11	0.48	0.02	0.35	0.08
22:1n-9	0.20	0.01	0.24	0.02
22:4n-6	0.08	0.02	0.63 *	0.07
22:5n-6	0.25	0.04	0.60 *	0.04
22:5n-3	2.80	0.09	2.20	0.78
22:6n-3	20.45 *	0.64	13.99	0.21

* denotes the significantly difference between two groups ($p < 0.05$). S.D., standard deviation

Reference

1. Ferosekhan, S.; Xu, H.; Turkmen, S.; Gómez, A.; Afonso, J.M.; Fontanillas, R.; Rosenlund, G.; Kaushik, S.; Izquierdo, M. Reproductive performance of gilthead seabream (Sparus aurata) broodstock showing different expression of fatty acyl desaturase 2 and fed two dietary fatty acid profiles. *Scientific Reports* **2020**, *10*, 15547, doi:10.1038/s41598-020-72166-5.
